# Understanding the Profile and Needs of Abused Men: Exploring Call Data From a Male Domestic Violence Charity in the United Kingdom

**DOI:** 10.1177/08862605211028014

**Published:** 2021-06-28

**Authors:** Benjamin Hine, Sarah Wallace, Elizabeth A. Bates

**Affiliations:** 1 University of West London, Brentford, UK; 2 University of South Wales, Pontypridd, UK; 3 University of Cumbria, London, Carlisle, UK

**Keywords:** domestic violence, intervention/treatment, disclosure of domestic violence, male victims, abused men

## Abstract

Current understandings on service engagement by male victims of domestic violence and abuse (DVA) within the United Kingdom (UK) have generally been captured by qualitative research. As such, large-scale quantitative data detailing the profile, needs and outcomes of abused men, upon both presentation and use of services, is currently lacking. The present study analyzed the client data of 719 callers to a domestic abuse helpline for men in the UK. Findings showed that the overwhelming majority of callers reported they were abused by female perpetrators, most of whom were still their current partner, and that many of the men were fathers. Vulnerable populations (GBTQ+ and disabled men) were under-represented in the sample. Most men were seeking emotional support, along with a range of practical advice and signposting to other services. The confidentiality of the helpline was crucial for many men, and almost half had struggled to access the service (suggesting a severe lack of resourcing). Findings are discussed in relation to the need for gender-inclusive services, which cater for the unique challenges and barriers experienced by abused men.

## Introduction

Male victims of domestic violence and abuse (DVA) have been chronically overlooked and have thus been termed a “hidden” victim population. This is partly the symptom of a dominant narrative across academic and societal discourse, which has framed DVA as something unilaterally perpetrated by men toward women as a function of patriarchal structures (Hine, 2019); the so-called “gender perspective” (Felson, 2002). However, research from the opposing “violence perspective” (Felson, 2002), and government statistics, have evidenced the existence of male victims of DVA for decades (Cook, 2009), and in considerable numbers. In 1975, the United States National Family Violence Survey sought to gather information to test causal influences of family violence, and was followed in 1985 by the second Family Violence Survey which was designed to capture how families coped with violence and the impact on physical and mental health (Straus & Gelles, 1995). Findings from both surveys revealed very similar perpetration rates among male (12%) and female (11.6%) partners (Straus et al., 2006). As a result, the terms “gender symmetry” and “gender asymmetry” became widely recognized in the 1980s (Straus et al., 2006), and research has since continued to emerge demonstrating that men can indeed be victims, and women perpetrators. This body of work culminated in the publication of a meta-analysis of 82 studies (and a total of 64,000 participants) that demonstrated that women were perpetrating physical aggression at rates of equal to or in fact significantly higher than men (with an effect size of *d* = –.05; Archer, 2000). This led to further work recognizing the prevalence of bidirectional or mutually violent relationships (Langhinrichsen-Rohling et al., 2012), the overlap of DVA and other types of violence (Bates et al., 2014), and the similarity of risk factors for men’s and women’s intimate partner violence (IPV) perpetration (Mederios & Straus, 2006).

Subsequently, research working with male victims is burgeoning. As a result, what was once considered a crime perpetrated solely by men toward women is increasingly being recognized (and evidenced by research) as also being perpetrated by women toward men. This is supported by recent figures from the Office of National Statistics (ONS), which show that an estimated 2.3 million adults (1.6 million women and 757,000 men aged 16-74 years) experienced DVA in the year ending March 2020 (Office for National Statistics, 2020). As such, increasing numbers of studies have since identified the severity and substantial range of abuse experienced by men, paralleling research on female victims; from physical aggression (Drijber et al., 2013; Hines et al., 2007) and psychological abuse (Bates, 2020), including coercive control, to sexual (Hines & Douglas, 2016b; Weare, 2018) and financial abuse (Hine et al., 2020). Moreover, unique vulnerabilities for male victims, including the use of legal and administrative aggression (Hines et al., 2015; Tilbrook et al., 2010), manipulation of parent-child relationships (Bates, 2019a; Bates & Hine, 2021; Hine, in press; Hines et al., 2007), and false allegations (Bates, 2020) have also been highlighted. Research utilizing gay, bisexual, and transgender (GBT) men has identified further forms of abusive behaviors, for example the use of HIV status and “outing” to control victims, and the deliberate misuse of pronouns (Barnes & Donovan, 2018). Taken together, while continuing inquiry is still needed (Morgan et al., 2014), work focusing on the experiences of male victims is beginning to develop into a significant body of research.

The impact of abuse on men has also begun to be explored in more detail, with studies demonstrating that DVA has demonstrable and long-term adverse impacts on the physical and mental health of both men and women (Alejo, 2014; Coker et al., 2002, 2000). Indeed for men, long-lasting negative consequences for overall physical (Hines & Douglas, 2015, 2016a) and mental health (Bates, 2019b) have been identified, including a higher prevalence of binge drinking (Hines & Straus, 2007) and posttraumatic stress disorder (PTSD; Hines & Douglas, 2011), in male victims, and, in GBT men, substance use and misuse (Bacchus et al., 2017). Importantly, for male victims who were also fathers, many report that the relationship with their child(ren) is affected, for example through experiences of alienation, parental relationship disruption, and the legal aggression mentioned above (Bates, 2019b). Moreover, this use of systems, particularly family courts, had a substantial impact on the mental health of male victims (Berger et al., 2016; Hine, in press; Hine & Bates, 2021). Indeed, family courts continue to be utilized as an avenue for abusive behaviors toward both men and women, and further investigation of the role of such systems in abusive contexts is desperately needed.

As a result of this body of research, it is fair to characterize abused men as “same-but-different” to abused women, in that, they appear to share many experiential characteristics, risk factors, and outcomes, which are then shaped or in some cases exacerbated in a gender-specific manner. For example, while research indicates that abuse is similarly impactful on men and women, they appear to employ contrasting externalizing and internalizing coping mechanisms respectively in response to this abuse. Similarly, while both men and women share concerns for their children’s welfare at the hands of abusive partners, men may experience additional barriers to exiting abusive settings, as their role in child-rearing is underestimated and provision for abused men to flee settings with their children is unavailable.

In light of this characterization, examination of men’s help-seeking behavior, and effective ways to provide support, has received some attention, with most studies highlighting the detrimental impact of gender stereotypes (Huntley et al., 2019). For example, in interviews with male victims, traditional masculine norms (i.e., that men should be strong, stoic, dominant, in control of their emotions, and able to cope on their own; Connell, 2005) had a significant impact on how men viewed themselves as victims, or whether they even recognized their victimization at all (Bates, 2019b; Machado et al., 2016). It should be noted that feelings of shame, humiliation, and embarrassment as barriers to recognizing abuse and help seeking are not unique to male victims and are frequently cited reasons for not reporting irrespective of gender (Thaggard & Montayre, 2019). However, aspects such as regressive gender norms, and how DVA is typically understood and framed as a heteronormative experience (Hine, 2019), serve to exacerbate these feelings for men. Indeed, the language around victimization is incredibly complicated for abused men, as they simultaneously grapple with the desire to resist such labels, while working toward recognition in order to effectively access and engage with services.

Such stereotypes are also reflected in reactions from others upon disclosure, with men reporting not being believed, being ridiculed, and describing how some services were mocking of their experiences, or suggesting they were somehow responsible for the abuse (Bates, 2019b). Indeed, men’s victimization is often assumed to be provoked in some way (Bates, 2020), as individuals seek to understand why women’s would go against their gender normative behavior and be aggressive (Scarduzio et al., 2017). It is important to acknowledge that female victims of abuse also face disbelief (Epstein & Goodman, 2018), particularly when from minority backgrounds or cultures, which normalize DVA against women (Burman et al., 2004), and that provision of belief and validation is recognized as important for all victims (Bates et al., 2001). However, men’s accounts appear almost unanimously reflective of such concerns, and they frequently describe how they are fearful they will not be taken seriously by authorities (Drijber et al., 2013), as demonstrated in work with men who have reported to the police (McCarrick et al., 2016). GBT men experience additional stigma, related to regressive beliefs around sexuality (Calton et al., 2016; Laskey et al., 2019), which is represented in their negative experiences of help-seeking (Donovan & Barnes, 2019), including reporting to law enforcement (Finneran & Stephenson, 2013), and the difficulty they face in accessing specialist services (Stiles-Shields & Carroll, 2015). This has led to the conclusion that that the quality of service provision for male victims is, at best, mixed (Bates, 2019b; Huntley et al., 2019).

Indeed, a recent review of victim services within the UK and United States has revealed that men remain an “underserved” population with fewer services available (including within the GBT community), great challenges associated with access to these services, and fewer empirical evaluations of effective provision for men (Bates & Douglas, 2020). For example, for female victims of DVA in England, a free 24-hour helpline exists, which answered over 108,000 calls in 2018-19 (Refuge, 2019). This is in contrast to the two most well-known helplines in England for male victims, which are open between 6 and 11 hours a day, presumably due to funding restraints or a perceived lack of need. Similarly, statistics collated by the Mankind Initiative reveal that in the UK there are currently 37 organizations that offer shelter and refuge space for men, which includes 204 spaces with 40 of these dedicated specifically for men only (Mankind Initiative, 2020). In contrast, for women there are currently 269 organizations and 3,649 spaces (Parliamentary Select Committee, 2017). Importantly, the proportionality of these figures is in stark contrast to available statistics around prevalence of victims by gender.

Recent research has sought to explore the experiences of service providers supporting male victims in an attempt to understand the challenges and barriers to effective service provision. For example, in their research with DVA services in Wales, Wallace et al. (2019b) highlighted how abused men faced a “tide of recognition,” which hindered men’s ability to accept and recognize their abuse and come forward. Service providers further explained that low numbers of men coming forward then undermined the evidence of need required to secure service funding, which, in turn, made provision of support difficult (with such challenges arguably reflective of sector-wide funding issues; Ishkanian, 2014). Such concerns are reflected in work with call-handlers in a UK based charity, with staff again highlighting that a lack of recognition for male victims (fueled by stereotypes about both men and domestic abuse) directly resulted in a lack of resourcing, which hindered the ability to provide quality support (Hine et al., 2020). Crucially, this study highlighted that service availability then acts as a significant barrier to developing further research around men’s service user experiences, as a lack of information on the prevalence and experiences of male victims, and a lack of service provision and support, mutually inform one another. This can best be described as a negative, self-fulfilling cycle, resulting in a lack of understanding within both academic and practitioner literature on how best to engage men, and what effective provision looks like for them as a population. It could therefore be argued that, if data were to be made available that demonstrated both the scale and scope of need in relation to abused men, this would provide both compelling and much needed direction and urgency for policymakers and funding authorities.

At present, little to no data on service engagement by abused men in the UK exists, largely due to the issues outlined above. The present study therefore analyzed case data provided by a UK domestic abuse helpline for men; described as providing a “confidential helpline…available for male victims of domestic abuse and domestic violence across the UK who are experiencing this abuse from their current or former wife or partner (including same-sex partner).” Case information included the demographic characteristics, abuse profile, caller needs, and call information and outcomes for callers accessing the helpline between August 2019 and March 2020. The study had one aim; to explore caller data in each of the four areas outlined above, to provide an assessment of caller profile and associated needs of male victims of DVA.

## Method

The data for the present study was provided by a UK domestic abuse helpline for men (known henceforth as Charity A), and collected by a larger, nationwide charity in the United Kingdom dedicated to ending DVA for all persons (known henceforth as Charity B). Charity A processes approximately 1,400 calls per year, both from male victims and those concerned about them (i.e., family and friends). They also receive over 200 calls a year from the police, councils, other support services and those in the legal profession. Charity B provides training for other DVA services that deliver frontline support to victims and is therefore described as an organization that designs and helps to deliver multiagency responses to DVA, both through their close work with other agencies and direct engagement with victims themselves. In this position, Charity B gathers nationwide data on DVA through a dedicated portal, collected from victims by service providers upon engagement with, and exit from, frontline DVA services. Between August 2019 and March 2020, a new module for this portal was utilized by Charity A to gather client data as part of a trial period to test the suitability of the portal for ongoing use. At the end of each call, call handlers asked if callers were happy to have their data collected for use by both charities. From a total of 1,402 callers, 727 (51%) agreed to complete the questionnaire. Some participants opted to fill out the questionnaire but did not wish their data to be shared; this was retained by Charity A but has not been used for analysis within this study. If callers were too distressed, or uncomfortable answering specific topics, they were not asked to complete the survey/specific questions, and call handlers used their best judgment in this matter. Call handlers also used their best judgments as to how reported behaviors should be coded, loosely using the descriptions under Table 3 to guide this process. Due to the confidential nature of the helpline, safeguarding measures are often not possible; a source of frustration highlighted by call handlers in previous research (Hine et al., 2020). Information on the demographic background of call-handlers, including training received, can be found in Table 1.

**Table 1.** Call Handler Demographic Information.

Pseudonym	Sex	Age	Role Within Organization (Alongside Call Handler)	Hours Worked (Per Week)	Time at Organization	Time in Sector Prior (Domestic Violence Support)	Qualifications/Training
Abagail	Female	58	Service manager	40	14 years	N/A	IDVA^a^, safeguarding^b^, supporting male victims^c^
Angela	Female	52	None	26	2 years, 6 months	N/A	Safeguarding, supporting male victims
Amy	Female	43	Head of training	26	21 months	N/A	IDVA, DVSM^d^, safeguarding, supporting male victims
Trish	Female	69	None	19.5	10 years	20 years (Set up first male victim refuge in England)	Safeguarding, supporting male victims

*Note.* ^a^Independent Domestic Violence Advocate.

^b^External Training Package.

^c^Internal Training Package.

^d^Domestic Violence Service Management.

A comprehensive data sharing agreement was constructed between Charity B and the lead author’s institution to ensure the correct and secure sharing of personal (anonymized) data. Indeed, when clients consented to provide their data, they acknowledged that the charity were free to share this data with third parties for the purposes of providing insight and to improve the experiences of victims. This study was approved by the University Research and Ethics Committee (UREC) at the University of West London.

## Results

Descriptive information on callers to the helpline is provided below in four core areas: demographic profile, abuse profile, caller needs, and call information and outcomes. For most variables, valid numbers and percentages (excluding missing data) are provided. *N* values for missing data are given in brackets at the end of each variable description. Only a small number of questions had a missing value frequency which exceeded 10% of the overall sample.

### Demographic Profile

In total, 727 caller case files were generated. Three of these were either a female caller (*n* = 2) or of an unknown gender (*n* = 1), so were excluded. A further five cases were excluded as the person was either calling on behalf of a victim (*n* = 1) or this information was unavailable (*n* = 4). This left 719 male callers who identified themselves as victims of abuse. The overwhelming majority (95.1%) reported a female abuser, whereas 34 (4.9%) reported a male abuser (*n*_miss_ = 19; Table 2). 96.5% of callers identified as heterosexual (with 23, or 3.3% identifying as gay, and 1, or 0.1% identifying as ‘Other’; *n*_miss_ = 27). Callers were also from a largely White background (560, 84.2%), with Asian (58, 8.7%) and Black (37, 5.6%) callers constituting the next largest backgrounds (followed by “Other Ethnicity,” *n* = 6, 0.9%, and “Mixed Ethnicity,” *n* = 4, 0.6%; *n*_miss_ = 54). Age data was available for 631 callers (*n*_miss_ = 88), showing clients to be aged between 20 and 76 years old, with an average age of 41 (*SD* = 10.84).

Most callers were in full-time employment (*n* = 510, 78%), with unemployed (*n* = 86, 13.1%), retired (*n* = 26, 4%), self-employed (*n* = 14, 2.1%), and being in Education or Training (*n* = 13, 2%) constituting the next highest percentages. A small number were stay-at-home parents (*n* = 3, 0.5%), were employed part-time (*n* = 1, 0.2%) or chose “other” (*n* = 1, 0.2%; *n*_miss_ = 65). The majority of callers either declined to say, or were not asked, about their financial situation (*n*_miss_ = 706). Of those that did provide this information, 11 said they had significant financial problems, 1 said they were managing essentials but had nothing left over, and 1 said they had no financial concerns. In total, 21 callers (3%) reported having a disability of some kind (*n*_miss_ = 16). In total, 207 callers reported that no children were “involved” in the abuse (i.e., they were not in the same household; 32.1%). In total, 182 callers reported one child in the house (28.2%), 173 reported two children in the house (26.8%), and 83 reported there being three children or more (*n* = 83, 12.9%; *n*_miss_ = 74).

**Table 2.** Demographic Profile.

	*n*	Valid %	*n*_missing_
Perpetrator gender			19
Male	34	4.9	
Female	666	95.1	
Sexual orientation			27
Heterosexual	668	9.5	
Gay	23	3.3	
Other	1	0.1	
Ethnicity			54
White	560	84.2	
Black	37	5.6	
Asian	58	8.7	
Mixed	4	0.6	
Other	6	0.9	
Employment status			65
Unemployed	86	13.1	
Retired	26	4.0	
Full-time employment	510	78.0	
Part-time employment	1	0.2	
Self-employed	14	2.1	
Education/training	13	2.0	
Say-at-home parent	3	0.5	
Other	1	0.2	
Disability			16
Yes	21	3.0	
No	682	97.0	
Age (yr)	*M* = 41.19, Min = 20.00, Max = 76.00, *SD* = 10.84		

### Abuse Profile

In total, 489 callers (68.1%) identified their abuser as their current intimate partner and 213 callers (29.7%) identified their abuser as an ex-intimate partner (Table 3). This means that 97.8% of callers were calling in reference to IPV, rather than familial violence. Other abusers identified were biological children (*n* = 5, 0.7%), step-children, brothers, other family members, fathers, other known persons (each *n* = 2, 0.3%), and mothers (*n* = 1, 0.1%; *n*_miss_ = 1).

In relation to types of abuse reported, the most frequent was psychological abuse, reported by 588 callers (81.8%). Physical abuse (*n* = 475, 66.1%), jealous and controlling behavior (*n* = 346, 48.1%) and financial abuse (*n* = 230, 32%) were the next most common. Some callers also reported sexual abuse (*n* = 14, 1.9%). Callers frequently reported more than one type of abuse, with just under half of callers reporting two abuse types. In terms of which abuse types co-occurred, cross tabs were calculated to assess how frequently any two abuse types co-occurred. While sexual abuse rarely co-occurred with any other abuse type, the highest co-occurrence was between physical abuse and psychological abuse (52.2% of the sample reported both abuse types). Other co-occurrences of note were jealous and controlling behavior, and psychological abuse (33.4%) and physical abuse (28.2%). Financial abuse was also often co-reported alongside physical (22.5%) and psychological abuse (23.9%). Abuse had been occurring for an average of 6.45 years (mean) before the call was made (*SD* = 5.91), and this ranged from very recently (<1 year) to a significant period of time (40 years). The median value for abuse length was five years.

**Table 3.** Abuse Profile.

	*n*	Valid %	*n*_missing_
Relationship			1
Current partner	489	68.1	
Ex-partner	213	29.7	
Mother	1	0.1	
Father	2	0.3	
Biological child (over 18)	5	0.7	
Step-child (over 18)	2	0.3	
Brother	2	0.3	
Other family member	2	0.3	
Other person/associate	2	0.3	
Abuse type			0
Physical^i^	475	66.1	
Sexual^ii^	14	1.9	
Jealous and controlling behavior^iii^	346	48.1	
Psychological^iv^	588	81.8	
Financial^v^	230	32.0	
Abuse (sum)			0
0 types	3	0.4	
1 type	96	13.4	
2 types	355	49.4	
3 types	214	29.8	
4 types	50	7.0	
5 types	1	0.1	
Abuse length (yr)	*M* = 5.78, Min ≤ 1, Max = 40.00, *SD* = 5.92		

^i^Coded if any physical injuries or physically abusive behaviors were described by callers, ranging from slapping to assault with a weapon.

^ii^Coded for abuse involving a sexual element, ranging from unwanted verbal approaches and/or sexual activity, to lying about pregnancy/miscarriage.

^iii^Coded if a caller described manipulation of their behavior by their partner, for example threats and false allegations, and controlling contact with others. This also included specific behaviors such as parental alienation.

^iv^Coded if verbal or degrading behaviors were described, including specific examples such as “gaslighting.”

^v^Coded for behaviors like controlling the finances of the caller, the abusive partner spending freely and putting the couple/abuse into debt, or expecting the abused partner to cover all outgoing expenses.

### Information and Signposting Needs

Out of the four types of information given, the most popular were emotional support (*n* = 678, 94.3%), signposting to other services^1^ (*n* = 650, 90.4%), and information/general advice (*n* = 511, 71.1%; Table 4). Very few clients required referral to other general agencies (*n* = 2, 0.3%). When examining the type of services clients were then signposted toward, the most popular were information about a solicitor (*n* = 457, 63.6%), the GP (*n* = 427, 59.4%), or the police (*n* = 391, 54.4%). Others included: information about community services (*n* = 187, 26.0%), child social services (*n* = 140, 19.5%), and other domestic abuse services (*n* = 135, 18.8%). Less frequent needs were as follows: housing services (*n* = 45, 6.3%), referral to a counsellor (*n* = 35, 4.9%), financial services (*n* = 36, 5.0%), mental health services (*n* = 21, 2.9%), alcohol misuse services (*n* = 14, 1.9%), other children’s services (*n* = 11, 1.5%), educational services (*n* = 7, 1.0%), immigration services (*n* = 4, 0.6%), physical health services (*n* = 2, 0.3%), adult social services (*n* = 2, 0.3), drug misuse services (*n* = 1, 0.1%), sexual violence services (*n* = 1, 0.1%), employment services (*n* = 1, 0.1%), or other (*n* = 57, 7.9%). Disability services, vocational training services, and other online services were not requested/needed by any callers.

**Table 4.** Information and Signposting Needs.

	*n*	%
Information given		
General advice	511	71.1
Signposting	650	90.4
Referral to other agency	2	0.3
Emotional support	678	94.3
Signposting		
Housing	45	6.3
Physical health services	2	0.3
Disability services	0	0.0
Mental health services	21	2.9
Drug misuse services	1	0.1
Alcohol misuse services	14	1.9
Child social services	140	19.5
Other children’s services	11	1.5
Other domestic abuse services	135	18.8
Sexual violence service	1	0.1
Adult social services	2	0.3
Financial services	36	5.0
Employment services	1	0.1
Educational services	7	1.0
Vocational training services	0	0.0
Community services	187	26.0
Immigration services	4	0.6
Online services	0	0.0
Police	391	54.4
GP	427	59.4
Solicitor	457	63.6
Counsellor	35	4.9
Other	57	7.9

### Call Information and Outcomes

The average length of calls made was 47 minutes (min = 3, max = 148, *SD* = 15.41; Table 5). Of concern, was that half of callers had tried contacting the helpline before and had not been able to get through (*n* = 345, 50.1%); this was not an issue for 343 callers (49.9%; *n*_miss_ = 31). When asked what alternative action they may have taken had they not made their call, 266 (36.9%) were not sure or did not know, 149 (20.7%) simply said they would keep looking and only 3 (0.4%) had a concrete plan, such as calling another helpline (*n*_miss_ = 301, 41.8%). Most callers found the helpline through a search engine (*n* = 400, 58.1%), with others finding the helpline through a mixture of routes (*n* = 291, 41.9%; *n*_miss_ = 30), including friends, family, colleagues, hospital staff, GPs, a counsellor, the police, a solicitor, and victim support.

In total, 711 callers (100%) described the call as useful (*n*_miss_ = 8), and 688 (99.7%) reported that they now knew where they could get help following the call (*n*_miss_ = 29). In total, 690 (99.9%) stated that they understood what options were available to them following the call (*n*_miss_ = 28), and 697 (99.1%) of callers stated that they felt better now that they had told someone (*n*_miss_ = 16). Interestingly, 418 callers (65.1%) stated that they would *not* have called had the helpline not been confidential (*n*_miss_ = 77, 10.8%).

**Table 5.** Call Information and Outcomes.

	*n*	Valid %	*n*_missing_
Tried to call before?	345	50.1	31
Alternatives to call			301
Do not know	266	36.9	
Keep looking	149	20.7	
Concrete plan (i.e., another helpline)	3	0.4	
How was helpline found?			30
Website	5	0.7	
Search engine	400	58.1	
Television	3	0.4	
Other (including family, friends, GP, etc.)	281	40.8	
Length of call (minutes)	*M* = 47, Min = 3, Max = 148, *SD* = 15.41)		
Call useful?	711	100	8
Now know where to go for help?	688	99.7	29
Understand options?	690	99.9	28
Felt better after call	697	99.1	16
Would not have called if not confidential	418	65.1	77

## Discussion

This study analyzed male victim caller data provided by a UK domestic abuse helpline supporting men. Case information including the demographic characteristics, abuse profile and context, caller needs, and call information and outcomes of callers accessing the helpline was examined. To our knowledge, this is the first study in the UK to collect this type of data, the analysis of which has enabled a unique exploration and greater insight into male victim callers’ profile and their associated needs.

### Demographic Profile

Of the 719 male callers to the helpline, results show that 95% were calling regarding abuse experienced by a female; with 5% of calls related to a male abuser. This finding supports the assertion that DVA experienced by men can occur in both opposite- and same-sex relationships, as well as data from the Scottish Justice Survey (2019) that indicated for male victims of partner abuse the majority (88%) reported their perpetrator was female. This is also contrary to prevalent stereotypes around DVA, which position only men as capable of being abusers (Hine, 2019). Furthermore, most men (68%) were calling in relation to abuse experienced by their current intimate partner. These figures are higher than those provided in other service reviews, which show 18% of victims are in an intimate relationship with their abuser at the point of accessing a service (though it should be noted that this report reviewed high-risk, frontline services; Safelives, 2020a). Nonetheless, the report highlights that victims living with their abuser are significantly less likely to report to the police and will experience abuse for an average of six years before seeking help (Safelives, 2020a). Male victims are already less likely to report their victimization to the police due to fear of not being believed, not being taken seriously, or being assumed to be the perpetrator (Drijber et al., 2013; Dutton & White, 2013). Indeed, the findings of the present study suggest that barriers to reporting abuse may be more prevalent in men, in part due to the proportion of men still living with their abuser. Moreover, DVA from an ex-intimate partner was experienced by 30% of callers. The types of postseparation abuse experienced by men is extensive; examples provided by Bates (2019a) include verbal aggression, false allegations, coercive control, harassment, withholding child contact, and manipulating relationships with children. We know from previous literature working with female victims that DVA can continue beyond the end of the relationship (Humphreys & Thiara, 2003), and can involve an escalation of abuse (Brownbridge, 2006), stalking and harassment behavior (Logan, 2019), and manipulation of the parental relationship (Zeoli et al., 2013). The data from this current study supports this suggesting that, similarly to women, many men do still suffer postseparation abuse at the hands of ex-partners.

The majority of male callers to the helpline identified as White. However, the numbers of men calling from ethnic minority backgrounds was largely in line with national figures for ethnic minorities (Office for National Statistics, 2019b). This contrasts to findings for female victims that suggests those of ethnic minority backgrounds struggle to access support (Burman et al., 2004; Kulwicki et al., 2010; Yoshioka & Choi, 2005), and might suggest that the helpline in this study offers a safe, accessible space for men from minority backgrounds, perhaps due to the reassurance of anonymity. This finding suggests that the helpline is seen as accessible to men of all ethnicities, though the specific reasons for this, and further research more broadly on the needs and experiences of ethnic minority male victims is still needed. For example, issues compounding the abuse experienced by minority men, which also impacts service needs and use, including racism, conflicts between religion and sexuality, and issues of language (Hester et al., 2012).

In contrast, the number of male callers to the helpline identifying as GBTQ were low. These findings mirror those found by specialist LGBTQ+ domestic violence services (Magić & Kelley, 2018) and Safelives outreach data (3% of users; 2020a). However, these figures are lower than actual rates seen in victim data, for example, the ONS (2020) reveals that in the UK, 6% of Gay men and 12.2% of Lesbian women experienced abuse, and the figures for Bisexual men and women are higher at 7.3% (men) and 19.6% (women). No data was reported for transgender men or women. In comparison, figures for heterosexual men and women were lower (3.5% and 6.9%). Findings from our study therefore suggest that either (a) GBTQ men may be less likely to come forward and disclose abuse and/or have less opportunities to do so, (b) that professionals may be correctly identifying and referring/signposting GBTQ victims, and/or (c) that opportunities to ask GBTQ men about victimization were limited. Societal heterosexism, fears, or threats of “outing,” and concerns of a lack of service understanding are additional barriers faced by abused GBTQ men (Carvalho et al., 2011; Donovan & Barnes, 2019; Hester et al., 2012; Magić & Kelley, 2018), all of which suggest a need for support services to actively promote their provision to GBTQ men and demonstrate their understanding of the issues GBTQ male victims can face. However, in order to promote provision, there has to first be provision, yet, specialist support for lesbian, gay, bisexual, transgender, and queer (LGBTQ) victims is largely unavailable within numerous local authority regions in England and Wales (Magić & Kelley, 2019). Again, the anonymity of this helpline may have been beneficial in this respect, but clearly was not enough on its own to promote engagement with the service by GBTQ men.

There were also low numbers of callers reporting having a disability, which raises the question of how abused men with disabilities disclose their abuse and access support. Disabled people experience disproportionately higher rates of DVA, which is more severe and frequent than individuals without disabilities (Public Health England, 2015). Again, ONS (2020) data shows rates of abuse experienced by individuals with disabilities are higher (7.5% men and 14.7% women) compared to 3.2% of men and 6% of nondisabled men and women. Nevertheless, research with abused men who have disabilities is virtually nonexistent (Ballan, 2017; Ballan et al., 2017). Data from the Multi-Agency Risk Assessment Conferences (MARACs), a meeting where information is shared on the high-risk domestic abuse cases^2^ between representatives from a range of agencies to increase victim safety and develop a coordinated action plan (Office for National Statistics, 2019a), supports the need for more research on why there appears to be under-representation from specific victim groups. For example, between April 2019 to March 2020, the number of cases heard at MARACs in the UK was 104,457 of which 15.2% were from a Black, Minority, Ethic (BAME) background, 1.3% were LGBT, and 6.5% of victims had a disability (Safelives, 2020b). Arguably, further research is needed within male victim populations on the experiences and needs of these groups, to address intersectionality with protected characteristics and aid provision of effective support.

Callers to the helpline were aged between 20 and 76 years with a mean age of 41 years. These findings echo those of Hines et al. (2007) where the mean age of callers was 41.32 years. Similarly, in Huntley et al.’s (2019) systematic review of the help seeking experiences of male victims of DVA, the typical age of men recruited to studies was between 40 and 60 years. These findings may suggest that men take a considerable amount of time to disclose their abuse. Indeed, findings from the current study show that prior to accessing the support of the helpline, male victims had experienced abuse for an average of five years, with one male caller experiencing 40 years of abuse. This mirrors findings from studies with male victims of sexual violence, which suggest that it takes longer for men to recognize and label their experiences as abuse (Easton, 2012; Walker et al., 2005). These results also build upon findings that, for men and women in England and Wales accessing services for high-risk individuals, it takes on average three years to access support from a DVA service (Safelives, 2018, 2020a), suggesting that the delay seen in this study may be particularly relevant to men who are not labelled, or do not see themselves, as “high risk.”

The findings from this study also show it is important to acknowledge that men irrespective of age can, and indeed do, experience DVA. Furthermore, caution should apply to assumptions about age and help seeking amongst male victims. Previous studies report that abused men tend to access informal sources of support such as family or friends (Morgan & Wells, 2016; Safelives, 2019), rather than formal sources of support. However, when abuse is severe, men are more likely to seek support from either formal or informal sources (Ansara & Hindin, 2010; Drijber et al., 2013). Further research should therefore explore the accessibility of services as a function of abuse severity and victim age, which would also serve to address gaps in the research working with older male victims (Carthy et al., 2019).

The majority of callers to the helpline reported being in full time employment; previous research has suggested that this is one of the barriers to men accessing nine-to-five services (Wallace et al., 2019a). Furthermore, this may have associated financial implications creating a barrier to leaving an abusive relationship. For abused women, financial barriers typically refer to a lack of income, which necessitates reliance on access to other funding sources. However, for men, financial barriers are more likely to result from obligations to joint mortgages or tenancies which can make securing alternative accommodation difficult (Hine et al., 2020). Furthermore, implications exist regarding access to safe accommodation for male victims who are employed. With a shortage of refuges spaces available for men throughout the UK (Bates & Douglas, 2020), there is a likelihood that men who are allocated a refuge space face a difficult decision to leave their employment to access the safety of a refuge, which may be a considerable distance from their employment, but also their friends and family (including children). Data from 2010 indicated that for the Mankind Initiative helpline on at least 120 occasions a man decided not to access safe accommodation because it was geographically too far away (Mankind Initiative, 2020).

Decisions to leaving the abuse (and family home) are likely to be heavily influenced by the presence of children. Worryingly, 25% of callers reported one child in the home, 24% reported two children in the home, and 12% reported there being three or more children in the home. Previous research has highlighted male victims’ reluctance to report DVA and/or leave the abusive relationship for fear of losing contact with their children (Bates, 2020; Hines et al., 2007), and a desire to protect their children from their abusive partner (Bates, 2019a; Lysova et al., 2020). Indeed, protection of children has been shown to be pivotal in women’s decisions to end abusive relationships also (Moe, 2009), and in this sense, abused parents appear equally motivated by the desire to protect their offpsring from an abusive partner. However, men are likely to experience additional barriers in this regard, due to (a) the limited provision for men fleeing with children, as outlined above, and (b) institutional biases which may overlook men’s role as victims and caregivers, and the potential of mothers to be violent toward their children (see Hine, in press, for review). This is supported by ONS statistics, which suggest that when men do leave the home (as a result of abuse or otherwise) they are rarely the resident parent (only 3%; Office for National Statistics, 2013). Services should therefore be aware of the impact of parenting on abuse dynamics and help-seeking (Hine et al., 2020). These findings also reitterate the more general issue of children being exposed to and experiencing DVA, as it is widely accepted that children living with DVA are at greater risk of experiecing neglect, physical, and/or sexual abuse (Devaney, 2015) and the impact of exposure to DVA is well evidenced (Hughes et al., 2017; Kitzmann et al., 2003).

### Abuse Profile and Caller Needs

Male victims calling the helpline had experienced a range of abusive behaviors that included psychological, physical, financial, coercive control, harassment, parental alienation, sexual abuse, and false allegations. These findings are supported by previous research that has highlighted the extent of abuse experienced by men (Bates, 2019a, 2020; Hine et al., 2020; Hines et al., 2007; Hines & Douglas, 2010a, 2010b; Wallace et al., 2019a), and provide further evidence of the extent of DVA perpetrated toward men (specifically by women). Such findings further existing evidence that abuse toward men is prevalent, severe, and supports calls for urgent attention and provision within the sector.

Several types of support needs were also identified in response to this wide-ranging abuse. Most men calling the helpline required emotional support, which demonstrates the importance of providing male victims with the assurance that they will be listened to, believed, and have their experiences validated (Hine et al., 2020; Wallace et al., 2019a, 2019b). This is similar to the needs highlighted in research working with female victims (Bates et al., 2001). However, there is strong support for the additional importance of belief and validation in helping male victims accept and recognize their abuse, due to the added challenges of overcoming masculine stereotypes and restrictive characterizations of DVA (Hine et al., 2020). Indeed, knowing they are believed affords abused men feelings of psychological strength (McCarrick et al., 2016), and failure to do so can lead to increased social isolation (Morgan & Wells, 2016). Alongside other practical avenues of assisting, abused men clearly want and need to be listened to, respected, and supported.

### Call Information and Outcomes

Barriers to help-seeking for male victims are exacerbated by gender stereotypes and DVA norms; that DVA is perpetrated by, not toward, men (Bates et al., 2019; Hine, 2019). This could explain why two thirds of men stated they would not have called if the helpline had *not* been confidential, highlighting that men may be seeking a safe, nonjudgmental space to seek support as a result. Again, the stigma and shame present around DVA victimization is present for female victims also, and likely results in barriers to disclosure. Indeed, more research and information are needed that explores women’s experiences of accessing telephone support lines, and whether they feel comfortable doing so and/or report positive experiences. However, there is strong theoretical and empirical evidence available, which suggests that confidentiality is particularly important for male victims (Hine et al., 2020), due to additional societal stigma related to a compromise in masculine ideals upon victimization.

Other barriers to male victims help-seeking also include a lack of knowledge about where to go and who can help (Huntley et al., 2019; Wallace et al., 2019b), which highlights the importance of accessibility and promotion of DVA provision for men. Indeed, there is a shortage of inclusive visible campaigns and promotion of DVA services, and for victims who do not “fit” the heteronormative experience, promotional materials, featuring imagery, and language consistent with the gendered narrative, may feel exclusionary. The online visibility of the helpline is therefore clearly an important feature for access, as most callers reported finding details of the helpline through search engines, alongside other routes including informal sources such as friends, family, and agencies like health and the police. While it is encouraging that informal and formal support systems are aware of male DVA specialist provision, it also demonstrates the importance of professionals being able to confidently enquire and safely manage disclosures of DVA from men and know how and where to signpost/refer to. This is something that clearly requires improvement, as referrals to outreach services in England and Wales (irrespective of gender) from services like health, housing, social care, and mental health are historically low (5%; Safelives, 2020a—though it should be noted that many of the services from which this data are drawn work exclusively with women). Men knowing what is available and where to go is further reflected by findings in this paper whereby caller needs included “signposting to other services,” “information/general advice,” and referrals to other services included solicitors, General Practitioners, or the police, suggesting that, alongside emotional support, male victims also require help practical assistance and signposting, which allows them to access safety/enforcement (police) and practical services (solicitors).

Crucially, issues regarding funding and availability were evident through the finding that around half of callers had tried calling the helpline before and had not been able to get through. In this study, around 1,400 calls were made across seven months, which equates to approximately seven calls per day. While it is likely that some of the issues with caller access would be due to a higher frequency of calls at particular times or “pinch points” (i.e., at Christmas), the organization *Refuge* reports processing around 300 calls per day without issue (Refuge, 2019). This supports numerous studies that have highlighted that, while a dearth of funding for DVA services exists more both broadly (Ishkanian, 2014), this is a particular issue for services supporting men (Hine et al., 2020; Wallace et al., 2019b), and that subsequently the sector is still largely oriented toward female victims (Bates & Douglas, 2020). The current study contributes toward highlighting the importance of ensuring that helplines like the one in this study are available and sustainable, as almost all men reported that the call had been useful and that they now knew their options and where to get help.

### Recommendations for Best Practice and Future Research Directions

First and foremost, the results from this study strongly suggest that current provision for male victims of DVA is inadequate, as demonstrated by the disappointing yet unavoidable inability of this helpline to consistently respond to callers when required. There is therefore a desperate need for increased service provision for abused men, which at least *attempts* to reflect the proportionality of male to female victims as best estimated by currently available statistics. Whether this support should be provided within an incorporated system that provides support for all victims, or by delineated, parallel services specifically for men is largely moot, as, regardless of how they are provided, services simply need to be constructed in ways that are gender-inclusive, and which consider the gender-specific experiences and barriers common to abused men (Hine, 2019; Hine et al., 2020). It is clear that the helpline from which this data was drawn is highly effective in its provision. As such, we make the following recommendations for best practice when working with abused men, regardless of where this provision is situated:

1. Services should provide anonymity, at least at the initial stage, to enable men to come forward without fear of judgment or embarrassment (Huntley et al., 2019).
2. Services should ideally provide a “baseline” provision, which recognizes and caters for the many areas of overlap between the experiences and subsequent needs of various victim groups (i.e., their desire for belief, variety of abuse reported, emotional and practical support requirements).
3. Services should then also be trained in the gender-specific needs of men, including, but not limited to: the impact of gender stereotypes on recognition and disclosure of abuse; cultural and structural barriers relating to men’s desire to remain with their children; how stereotypes relating to domestic violence mask men’s visibility; gender-specific coping mechanisms; and risks associated with length of time before disclosure and ongoing relationship/contact with their abusive partner.
4. Such training should center intersectionality, and an appreciation how various victim characteristics coalesce to inform experience and support needs (i.e., cultural background, sexuality, identified gender).
5. Where the feminist and gendered model of DVA was crucial to developing our current knowledge on women’s experiences, and indeed what we know about DVA in the sector to date, there is a need to be more open to alternative explanations. By moving our understanding of DVA in a more gender inclusive direction, it will allow the opportunity to understand it within the context of each individual victim and their experiences. This latter approach would also allow a much more tailored approach to supporting all victims.

The practice recommendations above directly underpin our subsequent recommendations for future research. Specifically, while there is now a significant body of work that has explored men’s experience of DVA, gaps in knowledge persist. For example, the current study has demonstrated the heterogeneity of male victims who have called this helpline seeking support, including a significant range of cultural backgrounds and ages. Yet, the wider evidence base lacks more detailed exploration of the intersectionality of different protected characteristics, which may impact on men’s experience of abuse and help-seeking; there is still little exploring victimization experiences of men from BAME groups, older men, GBT+ individuals, and men with disabilities. This has further implications because the current study, as well as wider data available on engagement with services (e.g., Independent Domestic Violence Advocate [IDVAs], MARAC), demonstrates that these groups are often underrepresented. Data from Safelives (2020a) shows that clients who were engaged with outreach services were mostly women (95%), heterosexual (90%), did not have a disability (79%), and were White British or Irish (84%). An informed evidence base of the experiences and needs of these groups will allow service providers to better understand ways in which to reach out, provide support, and encourage engagement with their provision. Such research should seek to employ diverse methodologies (e.g., integrative, mixed methods approaches) to ensure that the prevalence, severity, and impact of abusive experiences toward men is appropriately captured. As the complex experiences and needs of abused men become more widely evidenced, such findings can be used to inform services, DVA and policy strategies, while strengthening the need for better resourcing and long-term sustainable funding to support men.

### Limitations

There were several limitations with the current research. First, the client data in this study is gathered from callers to a helpline, rather than those engaging with services face-to-face. Therefore, while the anonymity and ease of access afforded by the helpline has produced a uniquely large data set with associated insights, the current study tells us little of men’s engagement with frontline services typical of the sector (e.g., refuges, IDVA services). Future research may thus consider expanding upon existing research exploring men’s experiences with such services (Wallace et al., 2019b), if and when such provision is developed and delivered on a large enough scale. Second, though the sample size in this study is substantial, many men refused to have their information recorded. There may, therefore, be some element of self-selection bias within the dataset analyzed. This was largely unavoidable however, due to the aforementioned issues identified for male victims during help-seeking.

### Conclusion

Findings from the current study suggest that men who seek support from services in the UK experience a wide range of abuse, perpetrated overwhelmingly by their female partner for lengthy periods of time, and who are likely to still be with their current partner at the time of seeking support. Challenges in engaging vulnerable populations within a population already plagued by barriers to help-seeking have been identified, including GBTQ and disabled men, and those with children. Crucially, the provision of the male DVA helpline described in this study is a vital source of support for male victims; providing belief, validation, and guidance about other types of services available. Funding for other services, which draw upon the most successful elements of the service in this study to provide gender-inclusive support to abused men, is clearly urgently required.

## Availability of Data and Material and Coding Apparatus

Data and apparatus for this study are not available due to confidentiality and sensitivity issues.

## Notes

1. In this context, signposting involved providing clients with information about other services only. This is in contrast to referral, where the service provider themselves make the contact with the alternate service, of which only two clients were availed. https://asauk.org.uk/wp-content/uploads/2013/09/Referral-Networks-key-steps-to-effective-signposting-and-referrals.pdf

## Author Biographies

**Benjamin Hine**, PhD, is a senior lecturer in psychology in the School of Human and Social Sciences at University of West London. His research focuses on the perceptions and stereotypes surrounding intimate partner and sexual violence, specifically in how victim gender influences evaluations of severity, responsibility and so on.

**Sarah Wallace**, PhD, is a senior research fellow in the Faculty of Life Sciences and Education at the University of South Wales. Her previous research has explored the provision of services for male victims within Wales, and the influence of stereotypes on men’s understanding of abuse and decisions regarding help-seeking.

**Elizabeth A. Bates**, PhD, is a senior lecturer in applied psychology at the University of Cumbria. Her research predominantly explores the experiences of male victims of domestic abuse, including postseparation abuse, and the impact violence has on their physical and mental health.
